# Obesity Discrimination in the Recruitment Process: “You’re Not Hired!”

**DOI:** 10.3389/fpsyg.2016.00647

**Published:** 2016-05-03

**Authors:** Stuart W. Flint, Martin Čadek, Sonia C. Codreanu, Vanja Ivić, Colene Zomer, Amalia Gomoiu

**Affiliations:** ^1^Academy of Sport and Physical Activity, Faculty of Health and Wellbeing, Sheffield Hallam UniversitySheffield, UK; ^2^Centre for Sport and Exercise Science, Health and Wellbeing Institute, Sheffield Hallam UniversitySheffield, UK; ^3^Department of Psychology, Charles UniversityPrague, Czech Republic; ^4^Department of Psychology, University of BathBath, UK; ^5^Department of Psychology, University of MariborMaribor, Slovenia; ^6^University of AmsterdamAmsterdam, Netherlands; ^7^Department of Psychology, Radbound UniversityNijmegen, Netherlands

**Keywords:** obesity, discrimination, workplace, implicit, explicit

## Abstract

Previous literature reports that obese persons are discriminated in the workplace. Evidence suggests that obese people are perceived as having less leadership potential, and in comparison to normal weight peers, are expected to be less successful. This study examined whether obese people are discriminated against when applying for employment. Three hypotheses were offered in line with previous research: (1) obese people are less likely to be assessed positively on personnel suitability than normal weight people; (2) obese people in active employment are more likely to be discriminated against than people in non-active employment; and (3) obese women are more likely to be discriminated against than obese men. 181 Participants were sampled from sedentary, standing, manual and heavy manual occupations. Participants rated hypothetical candidates on their suitability for employment. Employees also completed measures of implicit and explicit attitudes toward obesity. MANOVA was conducted to examine if obese candidates were discriminated against during the recruitment procedure. Results demonstrated that participants rated obese candidates as less suitable compared with normal weight candidates and when the weight status of the candidate was not revealed for work across the four workplace groups. Participant gender and weight status also impacted perceptions of candidates’ suitability for work and discrimination toward obese candidates was higher in participants from more physically demanding occupations. The study findings contribute to evidence that obese people are discriminated against in the hiring process and support calls for policy development.

## Introduction

Worldwide prevalence of obesity has increased with approximately half a billion people currently classed as obese (Body Mass Index ≥ 30 kg.m^2^; World Health Organization, 2015). Concurrently, there has been an increase in the stigmatization and discrimination of obesity (Latner and Stunkard, 2003). A number of institutions contribute to the development and maintenance of anti-fat attitudes in particular the media, such as television or written press (Latner et al., 2007; Flint et al., 2016). The discourse used in the media when reporting on obesity represents an attempt to create concern and a ‘moral panic’ and in doing so leads to an emotional response in the recipient (Rich and Evans, 2005; Tischner and Malson, 2008). Indeed, the reach and influence of these institutions is wide and as such, obesity stigmatization has been reported in various population groups: jurors (Schvey et al., 2013), healthcare professionals (Carr and Friedman, 2005; Brown et al., 2007), educational professionals (Puhl and Heuer, 2009), and obesity researchers (Flint and Reale, 2014). Consequently, research has identified that there are settings where obesity stigmatization and discrimination may occur such as in schools (Puhl and Luedicke, 2012), public spaces including waiting rooms and public transport (Puhl and Brownell, 2001), and in the workplace (Flint and Snook, 2014).

Research investigating obesity discrimination in the workplace has examined the stereotypes reported about obese employees and how these may translate to discriminative behaviors. Both experimental and survey research suggest that employment discrimination against overweight individuals is substantial in Western cultures (see Roehling, 1999, for an extensive review). More specifically, Levine and Schweitzer (2015) found that people with obesity were associated with low competence, whilst Schulte et al. (2007) reported that obese people receive lower starting salaries, are ranked as less qualified, and work longer hours than normal weight employees. Similarly, Ball et al. (2002) suggested that obesity and high BMI are associated with employment in jobs associated with lower socioeconomic status. There are also reports of discrimination at the hiring stage, where obese candidates are assessed having less leadership potential, are less likely to be employed, and are expected to be less successful compared to normal weight peers (Flint and Snook, 2014). Moreover, Agerström and Rooth (2011) reported that managers held negative automatic stereotypes about obese people and were less likely to invite an obese applicant for an interview.

In the hiring process, a number of additional factors have been reported to have an impact on obesity discrimination, such as the candidate’s gender and the requirements of the job. Specifically, obese women were almost three times more likely to report discrimination than obese men (Roehling et al., 2007). Bartels and Nordstrom (2013) suggested that obese women are more likely to be discriminated against than obese men when applying for a job, especially if the job requires high visibility and physical demands. Previous research and theory, such as the Objectification Theory (Fredrickson and Roberts, 1997) suggests that unlike men, women are subjected to sexual objectification and assessment against beauty standards. Fredrickson and Roberts argue that the objectification of women is harmful to women and this explains why women are judged more harshly compared to men in many spheres of life including employment discrimination. It is suggested that people are socialized into objectifying women based on beauty standards with a plethora of examples evident in current society such as in the media.

Bartels and Nordstrom (2013) provide the most recent evidence regarding obesity discrimination in the hiring process whilst assessing gender and the physical requirements of employment. However, there were methodological shortcomings of Bartels and Nordstrom’s (2013) study that the current study aimed to improve on. First, Bartels and Nordstrom’s (2013) study only examined perceived suitability of a hypothetical candidate when weight status was revealed. Thus, the weight status of candidates was always revealed in their study which is not always the case when applying for employment and unlikely in countries where a picture of the candidate is not a required element of a CV. The current study aimed to compare the perceived suitability of candidates whose weight status was not revealed. Second, as acknowledged by Bartels and Nordstrom (2013), in their study participants rated only one potential candidate per position which is also unlikely in a real hiring process, where typically a range of candidates are assessed. Third, Bartels and Nordstrom (2013) only examined explicit anti-fat attitudes, despite previous research (e.g., Flint et al., 2015a) suggesting implicit measures are a strong predictor of anti-fat attitudes. Finally, only 44% of participants were employed and 45% had experience of recruiting employees in Bartels and Nordstrom’s (2013) study.

Unemployment is a longstanding topic of concern across the world, with research linking unemployment with poorer outcomes such as increased likelihood of health disparities (Adler and Newman, 2002). Reports of obesity discrimination in the hiring process have led to calls for the development and review of legislation to protect obese people from discrimination (Flint and Snook, 2015).

In 2014, the European Court of Justice ruled that being severely overweight could be considered a disability if it significantly disrupted an employee’s ability to work. In the US, there are states that have laws to protect against height and weight discrimination, whilst UK and EU employment law is lagging and is yet to include discrimination toward overweight and obese people. The main problem with the existing anti-discrimination laws is that they require interpretation of an individual’s (dis)ability to work, as well as of the necessary adjustments that may be required to accommodate a person’s needs (Flint and Snook, 2014). Anti-discrimination laws are included in legislation such as The UK Equality Act (2010) and EU laws in The Employment Equality Directive (2007/78). For example, The UK Equality Act (2010) specifically prohibits discrimination on the grounds of age, disability, gender re-assignment, marriage and civil partnership, pregnancy, maternity, race, ethnicity, religion, belief, sex, and sexual orientation. In considering current anti-discrimination legislation and thus protection for obese people from discrimination, whether obesity is defined as a disability is the key consideration. For example, obesity is not specified as a disabling condition in The UK Equality Act (2010). Thus, through interpretation, an obese person who experiences discrimination in recruitment or in the workplace such as being overlooked for a job or promotion may not be protected by current legislation. Consequently, misunderstandings and misinterpretations of obesity may lead to stereotyping and discriminative behaviors in the workplace.

The hiring process in employment is clearly an area that warrants further examination given that previous research suggests obese people experience discrimination when applying for work, and the implications of unemployment which continues to be a global concern. Thus the aim of the present study was to identify whether obese people are discriminated against when applying for employment and by doing so improve on the methodological limitation of previous research. In line with previous research identified above, three hypotheses were formulated: (1) obese candidates will be assessed as less suitable for employment than normal weight candidates; (2) obese candidates are more likely to be discriminated against when applying for employment in active working environments compared to non-active environments; and (3) obese women are more likely to be discriminated against than obese men.

## Materials and Methods

### Participants

Hundred and eighty one employees (107 male, 74 female) in employment varying in levels of physical demand (sedentary, standing, manual work and heavy manual work) took part in the study. All participants were fluent in English and sampled from three European countries: Czech Republic, Slovenia, and the UK. There was no compensation or incentives for participating in the experiment. Using a convenience sample of workplaces, employees responded to requests received via email for participation to take part in the study. Workplaces were selected that corresponded to the activity levels as stated in European Prospective Investigation into Cancer and Nutrition physical activity questionnaire (EPIC, Wareham et al., 2003). Participants had to have had previous experience of recruiting employees to the workplace.

### Materials

A range of implicit and explicit measures were used to assess anti-fat attitudes and beliefs about the controllability of obesity: Implicit Association Test (IAT; Greenwald et al., 1998), Attitudes Toward Obese Persons scale (ATOP; Allison et al., 1991), Beliefs About Obese Persons scale (BAOP; Allison et al., 1991), and F-scale (Bacon et al., 2001).

The IAT (Greenwald et al., 1998) is a computer-based measure of implicit attitudes which was modified in this study to assess attitudes toward fatness and thinness. Scores range between -2 and 2 with positive scores indicative of implicit anti-fat or pro-thin preference. The seven block IAT will be employed as described by Greenwald et al. (2003, see **Table 1**). The quicker participants assign stimuli to the grouping categories in blocks 4 and 7, the stronger implicit attitude toward the pairings. Previous research (e.g., Greenwald et al., 1998) has reported satisfactory internal consistency with Cronbach’s alpha ranging from of 0.7 to 0.9.

**Table 1:** Job suitability, implicit and explicit attitudes toward obesity for gender and workplace activity level (mean and standard deviation).

Measure		Gender		Workplace activity level			
*n* = 181 (107/74)	Overall	Male 107	Female 74	Sedentary 43 (19/24)	Standing 56 (35/21)	Manual 47 (24/23)	Heavy Manual 35 (29/6)
Male normal weight CV	39.14	39.73	38.28	38.88	38.29	38.83	41.23
	(2.80)	(2.44)	(3.06)	(3.33)	(2.85)	(1.94)	(1.91)
Female normal weight CV	34.65	34.19	35.32	37.47	34.34	34.81	31.49
	(2.93)	(2.86)	(2.93)	(2.96)	(2.14)	(1.60)	(1.82)
Male obese CV	25.38	24.63	26.47	29.88	26.00	24.21	20.43
	(4.12)	(3.95)	(4.14)	(3.39)	(2.82)	(1.96)	(2.05)
Female obese CV	23.31	22.39	24.65	29.51	24.89	20.51	16.94
	(5.26)	(5.24)	(5.04)	(3.33)	(3.06)	(2.18)	(2.44)
Male no photo CV	30.42	30.35	30.53	32.98	30.18	29.17	29.34
	(2.89)	(2.53)	(3.36)	(3.35)	(2.87)	(1.74)	(1.14)
Female no photo CV	28.27	27.75	29.03	31.88	29.02	27.45	23.74
	(3.73)	(3.93)	(3.29)	(3.02)	(3.11)	(1.32)	(2.17)
IAT	0.76	0.79	0.71	0.60	0.61	0.86	1.05
	(0.33)	(0.34)	(0.31)	(0.32)	(0.24)	(0.24)	(0.31)
ATOP	65.72	63.98	68.24	73.77	70.09	62.28	53.49
	(10.52)	(10.45)	(10.18)	(9.50)	(9.06)	(5.65)	(3.86)
BAOP	22.79	21.35	24.88	28.37	25.48	20.68	14.46
	(6.69)	(6.69)	(6.17)	(5.14)	(4.51)	(4.17)	(4.38)
F-scale	3.52	3.60	3.42	3.25	3.35	3.72	3.88
	(0.37)	(0.34)	(0.39)	(0.40)	(0.29)	(0.21)	(0.12)

*IAT, Implicit Association Test scores range from -1 to 1 with positive scores indicative implicit anti-fat/pro-thin bias; ATOP: Attitudes About Obese Persons scale. Scores range from 0 to 120 with lower scores indicative of negative attitudes toward obese persons; BAOP: Beliefs About Obese Persons scale scores range from 0 to 48 with lower scores indicative of stronger beliefs that obesity is controllable; F-scale: The Fat Phobia scale short form scores range from 1 to 5 with higher scores indicative of higher fat phobia.*

The ATOP (Allison et al., 1991) measures both positive and negative attitudes toward obese people. The scale has 20 items that are measured on a six-point Likert scale (+3 to -3) for each statement. Scores range from 0 to 120 with higher scores indicative of more positive attitudes toward obese persons. Previous research (e.g., Allison et al., 1991) has reported satisfactory internal consistency with Cronbach’s alpha scores ranging between 0.65 and 0.83.

The BAOP (Allison et al., 1991) measures the extent that an individual believes that obesity is under an individual’s control. The scale contains eight items that are measured on a six-point Likert scale (-3 to +3) for each statement. Overall scores range from 0 to 48 with lower scores indicative of a stronger belief obesity is controllable. Previous research (e.g., Allison et al., 1991) has reported satisfactory internal consistency with Cronbach’s alpha scores ranging from 0.80 to 0.84.

The F-scale (Bacon et al., 2001) measures the extent that respondents associate negative characteristics with being fat. The 14 item scale is measured on a five-point Likert scale where two opposing attributes are presented together (e.g., 1 = Active to 5 = Lazy). Previous research (Bacon et al., 2001) has reported satisfactory internal consistency with Cronbach’s alpha scores ranging from 0.87 to 0.91 in different samples.

Six hypothetical candidate CVs were developed (a male and female normal weight, obese, and no photo CV) that were modified for the four physical activity levels of the workplace as identified in the European Prospective Investigation into Cancer and Nutrition physical activity questionnaire (EPIC; Riboli et al., 2002). Thus, the hypothetical candidates were applying to four different employment offers (one for each physical activity level). For the sedentary workplace the advertised job offer was an administrative assistant, for the standing workplace a university lecturer, for the manual workplace a retail salesperson and for the heavy manual workplace a laborer. The advertised jobs were at early career level and thus CVs were a maximum of two pages in length to standardize across workplace.

All CV were developed to match the requirements of existing employment opportunities resulting in highly competent candidates. Thus, none of the hypothetical candidates could be rejected based on insufficient professional experience or skills. The content of the CVs was standardized including basic contact information, education, personal and professional experience with variation across four workplaces (e.g., academic CV had list of selected publication). CVs were randomly allocated and counterbalanced for gender and weight status such that each participant rated two normal weight CVs (one male, one female), two obese CVs (one male, one female) and two CVs without a photo (one male, one female). The sex of the participants was indicated by the name of the applicant. Common British male or female only names were used (i.e., no unisex names) to avoid any confusion regarding the gender of the CVs without a picture.

A Personnel Suitability scale was developed for the study comprised of seven items that aimed to measure the participants’ evaluation of the hypothetical candidates. Qualities assessed on a seven-point Likert scale included: team-work ability, social competence, job efficiency, intelligence, motivation, and leadership skills. One question explicitly inquired whether the candidate was considered to be suitable for the job. A cumulative score of all items was calculated, thus scores for the scale range from 0 to 42.

### Procedure

This study received institutional ethical approval from the Faculty of Health and Wellbeing, Sheffield Hallam University; Department of Psychology, Charles University; Department of Psychology University of Bath; and Department of Psychology, University of Maribor. All participants provided written informed consent in accordance with the Declaration of Helsinki.

Participants were recruited from four workplace environments that require different levels of activity (sedentary, standing, manual work and heavy manual work) as measured by the EPIC (Riboli et al., 2002). Each participant evaluated the job suitability of six hypothetical candidates based on their CV, before completing the IAT (Greenwald et al., 1998), the ATOP and BAOP (Allison et al., 1991), and the F-scale (Bacon et al., 2001). Participants only rated hypothetical candidates who were applying for employment in a workplace that corresponded with their own workplace. Demographic data about the participants was also collected. All participants rated the suitability of each CV prior to the implicit and explicit measures in order to avoid revealing the topic of enquiry to participants. The implicit and explicit measures were completed in a counter-balanced order. On completion of the test, all participants were debriefed regarding the full aim of the experiment.

### Analysis

A repeated measures Multivariate Analyses of Variance with Within-Subject gender (male, female) and photo (normal, obese, no photo) and between subject workplaces (sedentary, standing, manual and heavy manual) was conducted. Where significant interactions were found, follow-up ANOVAs were conducted. The model was a composite of Photo × Gender × Workplace. Repeated measures ANOVA was conducted to assess whether obese candidates were less likely to be assessed positively on personnel suitability scales than normal weight candidates. The test compared suitability scores of the photo condition (obese, normal, and no photo) as a within subject variable, and whether obese women are more likely to be discriminated against than obese men a repeated measures ANOVA was conducted.

## Results

### Descriptive Statistics

Participants were aged 24–60 years (*M* = 38.25, *SD* = 8.99) with a mean BMI of 25.9 kg.m^2^ (*SD* = 3.39). Age distribution was slightly left skewed with higher frequency of younger participants. Distribution across level of physical activity in the workplace was 43 in sedentary occupation (19 males), 56 in standing occupation (35 males), 47 in manual work occupation (24 males), and 35 in heavy manual work occupation (29 males). Mean scores on for the hypothetical candidates’ suitability for work demonstrated that both males and females perceived the normal weight male as the most suitable for employment (39.73 and 38.38 out of 42 respectively) and the obese female as the least suitable for employment (22.39 and 24.65 out of 42 respectively; see **Table 1**). Overall, participants reported negative implicit and explicit attitudes toward obesity and a belief that obesity is controllable. Male participants from the heavy manual workforce reported the most negative implicit and explicit attitudes toward obesity with lower ATOP and BAOP scores, higher IAT and F-scale scores (see **Table 1**). Participants sampled from the heavy manual workplace also reported the strongest beliefs that obesity is controllable than the other types of workplace (see **Table 1**).

**FIGURE 1:**
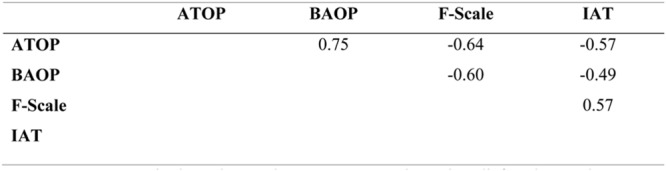
**Correlations between implicit and explicit measures.** ATOP, BAOP: Attitudes About Obese Persons Scale and Beliefs About Obese Persons Scale; F-Scale: The Fat Phobia Scale short form; IAT: implicit association test.

A number of correlations were apparent among the explicit measures (see **Figure 1**). A positive correlation was found between ATOP and BAOP scales indicating that more negative attitudes toward obese persons were associated with a stronger belief that obesity is controllable. A negative correlation was found between the ATOP and F-scale and the BAOP and F-scale meaning that stronger beliefs about the controllability of obesity and negative attitudes toward obese people are associated with greater fat phobia. There was also a positive correlation between the IAT and F-scale indicating an association between negative implicit anti-fat or pro-thin bias and greater fat phobia. Finally, negative correlations were observed between the IAT and ATOP, and IAT and BAOP, suggesting that more negative implicit anti-fat or pro-thin bias are associated with more negative attitudes toward obese people and stronger beliefs that obesity is controllable.

### Main Effects

Three statistically significant main effects and no statistically non-significant effects resulted from initial MANOVA. The largest effect size was measured for Photo [*F*(2,176) = 1950.97, *p* < 0.001, $\eta_{p}^{2}$ = 0.957], followed by Gender [*F*(1,177) = 381.82, *p* < 0.001, $\eta_{p}^{2}$ = 0.683] condition. Both of former variables are Within-Subject. Main effect was also observed among Between-Subject Workplace Setting variable [*F*(3,177) = 115.33, *p* < 0.001, $\eta_{p}^{2}$ = 0.662].

### Interactions

**FIGURE 2:**
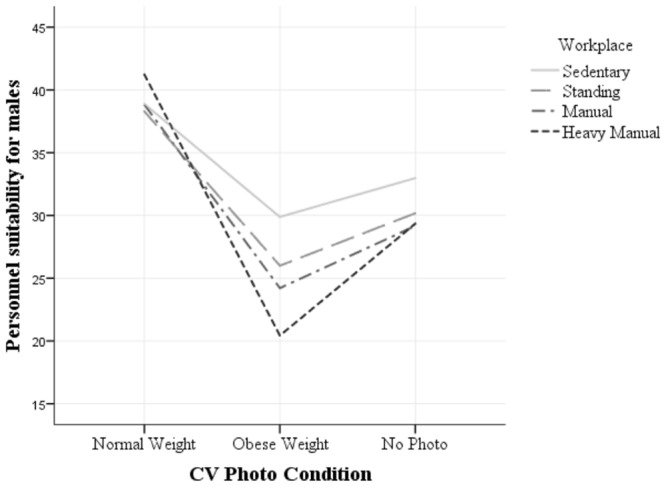
**Three Way Interaction of Gender × Photo × Workplace (male)**.

A significant three-way interaction was observed between Gender × Photo × Workplace [*F*(6,354) = 12.39, *p* < 0.001, $\eta_{p}^{2}$ = 0.17 see **Figures 1** and **2**]. There was also a significant two-way interactions of Gender × Photo [*F*(2,176) = 59.50, *p* < 0.001, $\eta_{p}^{2}$ = 0.40], Photo × Workplace [*F*(6,354) = 25.24, *p* < 0.001, $\eta_{p}^{2}$ = 0.30] and Gender × Workplace [*F*(3,177) = 44.90, *p* < 0.001, $\eta_{p}^{2}$ = 0.43]. These results demonstrate that the factors in the model interact significantly.

**FIGURE 3:**
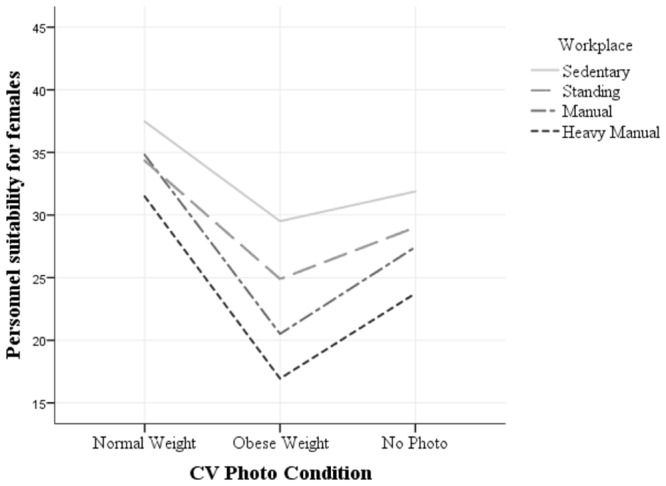
**Three Way Interaction of Gender × Photo × Workplace (female)**.

The three-way interaction shows that participants judged the personnel suitability of the CVs with significantly different scores depending on gender. Furthermore, the score was significantly different across each of the workplaces with heavy manual workplace interacting with the other workplaces. Hence, as the activity level of the workplace increased, stigma toward obese female candidates increased while normal weight male candidates were perceived as more suitable. The two-way interactions further confirm that male and female CVs were judged significantly different when photo conditions were manipulated, and that the CVs were judged differently based on the photo conditions across workplace settings. Finally, personnel suitability of the candidate was judged significantly different across all workplaces based on gender. These results also indicate that the manipulation has been effective (see **Figures 2** and **3**).

### Personnel Suitability

To test Hypothesis 1, follow up repeated measures ANOVA demonstrated that personnel suitability was judged significantly different across Photo condition [*F*(1.49,268.57) = 1249,40, *p* < 0.001, $\eta_{p}^{2}$ = 0.87]. Follow up pair-wise *t*-tests confirm that obese candidates are judged significantly less suitable than normal weight candidates [*t*(180) = 39.47, *p* < 0.001, Cohen’s *d_z_* = 2.94] and the No Photo condition [*t*(180) = 22.95, *p* < 0.001, Cohen’s *d_z_* = 1.71]. Also, normal weight candidates were judged significantly more positive than the No Photo condition [*t*(180) = 36.35, *p* < 0.001, Cohen’s *d_z_* = 2.70]. These results are in lines of Hypothesis 1 as obese candidates were assessed as less suitable for employment compared to normal weight candidates. A significant interaction between Gender and Photo was also evident [*F*(2,360) = 47,11, *p* < 0.001, $\eta_{p}^{2}$ = 0.21] and is further interpreted below is relation to Hypothesis 3.

### Workplace Activity Level

To assess whether obese people applying to active working environments are more likely to be discriminated against than in non-active working environment (Hypothesis 2), a repeated measures ANOVA using the averaged personnel suitability score (see **Table 1**) of gender between Photo conditions as within-subject factor and workplace as a between subject factor was conducted.

There was a significant interaction between Workplace and Photo [*F*(6,354) = 52.95 at *p* < 0.001, $\eta_{p}^{2}$ = 0.47]. Main effects were found for Photo [*F*(2,354) = 2380.55, *p* < 0.001, $\eta_{p}^{2}$ = 0.93] and Workplace [*F*(3,177) = 115.33, *p* < 0.001, $\eta_{p}^{2}$ = 0.662]. To further analyze the interactions, a one-way ANOVA was conducted for each workplace to examine differences between photo conditions. Significantly different judgments of suitability were reported for all four workplace activity levels, between Photo conditions: obese [*F*(3,177) 171.971, *p* < 0.001, η^2^ = 0.74]; normal weight [*F*(3,93.39) = 5.82, *p* < 0.001, η^2^ = 0.16]; and No Photo [*F*(3,91.94) = 58.46, *p* < 0.001, η^2^ = 0.66; see **Table 1**]. A Tukey *post hoc* test revealed significant differences across all workplace groups in the obese and No Photo conditions (*p* < 0.05). A significant difference in the normal weight condition was found between the sedentary workplace group compared to the standing, manual and heavy manual workplaces (*p* < 0.001).

The lowest average suitability score across all the groups was received by obese candidates, which decreased as the physical demands of the workplace increased. Candidates without a photo received higher suitability scores than obese candidates, however a similar trend in the obese photo condition was observed. Finally, normal weight candidates profited from including their photo in their CVs. Such candidates were rated significantly more suitable for heavy manual workplace. Their scores across the rest of workplaces were similar and on average, higher than the No Photo and obese conditions. These results are in support of Hypothesis 2; hence obese candidates applying for employment in active environments were discriminated more than in non-active environment.

### Gender Discrimination

**FIGURE 4:**
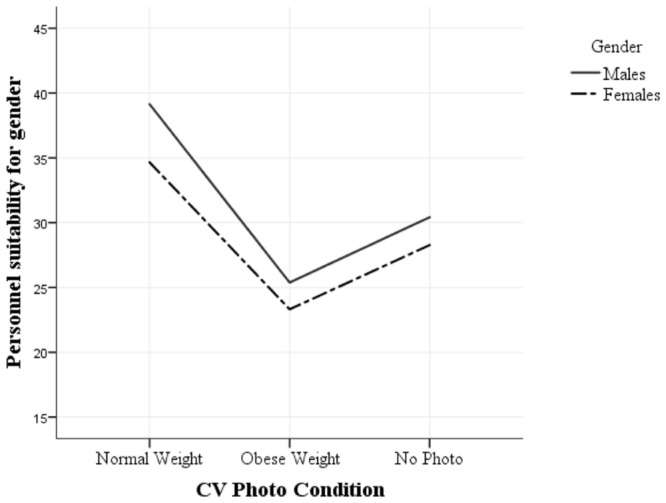
**Gender difference across obese, normal weight, and no photo conditions**.

To test Hypothesis 3, repeated measures ANOVA demonstrated that there was a significant interaction between Gender and Photo [*F*(2,360) = 47.11, *p* < 0.001, $\eta_{p}^{2}$ = 0.21]. A main effect for Gender and Photo was found [*F*(1,180) = 196.79, *p* < 0.001, $\eta_{p}^{2}$ = 0.52; *F*(1.49,268.57) = 1249.40, *p* < 0.001, $\eta_{p}^{2}$ = 0.87, respectively]. Pairwise comparisons revealed that males were assessed as more suitable that females (*M_diff_* = 2.90, *p* < 0.001, 95% CI [2.49, 3.31]; see **Figure 4**). These results support Hypothesis 3 that obese women are more likely to be discriminated against than obese men (see **Figure 4**).

**FIGURE 5:**
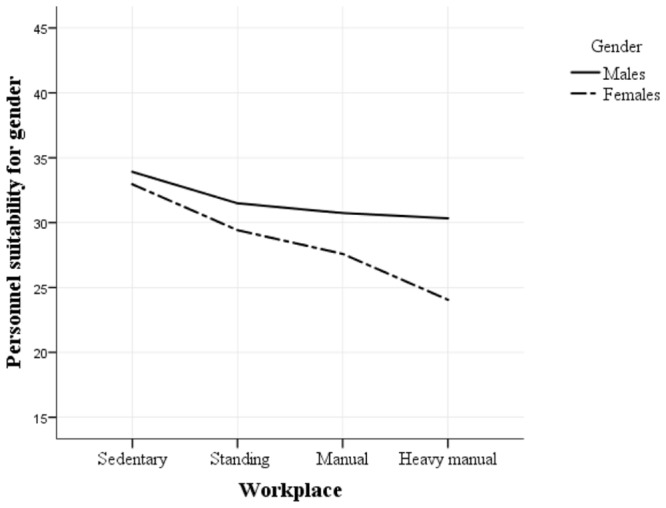
**Gender difference across workplace activity level**.

An significant interaction was evident between Workplace and Gender [*F*(3,177) = 44.90, *p* < 0.001, $\eta_{p}^{2}$ = 0.43]. The results are captured in **Figure 5**, where both males and females suitability scores reduce as the activity level of workplace increases. **Figure 5** demonstrates that this is more profound for females compared to males.

To assess the any gender difference in the gap between obese weight, normal weight and no photo conditions, paired sample *t*-tests was conducted. Significant differences were observed between normal weight and obese candidates [*t*(180) = 7.97, *p* < 0.001, Cohen’s *d_z_* = 0.59, 95% CI [1.82, 3.02]] and normal weight and No Photo candidates [*t*(180) = 8.60, *p* < 0.001, Cohen’s *d_z_* = 0.64, 95% CI [1.80, 2.87]]. No difference was observed between the No Photo and obese candidates [*t*(180) = 0.30, *p* > 0.05, Cohen’s *d_z_* = 0.02, 95% CI [-0.46, 0.62]]. These results indicate that there is a normal weight bias compared to obese candidates or when the weight of the candidate is ambiguous.

Finally, to examine whether the suitability scores were predicted by the participants’ attitudes and beliefs about obesity (i.e., scores from the ATOP, BAOP, F-scale, and IAT). Multivariate multiple regression demonstrated that BAOP [*F*(6,171) = 5,57, *p* < 0.001, $\eta_{p}^{2}$ = 0.164] and F-scale [*F*(6,171) = 3,82, *p* = 0.001, $\eta_{p}^{2}$ = 0.118] scores had a statistically significant relationship with the joint distribution of the suitability scores. Non-significant findings were evident for both the IAT [*F*(6,171) = 1,61, *p* > 0.05, $\eta_{p}^{2}$ = 0.053] and ATOP [*F*(6,171) = 1,23, *p* > 0.05, $\eta_{p}^{2}$ = 0.041]. Scores on the BAOP (belief that obesity is controllable) significantly predicted perceived suitability of the Normal Weight Male, Normal Weight Female, Obese Weight Male, Obese Weight Female, and Female candidate without a photo (*B* = -0.117, 95% CI [-0.207, -0.27], *p* = 0.01, adjusted *R*^2^ = 0.101, $\eta_{p}^{2}$ = 0.036; *B* = 0.177, 95% CI [0.090, 0.264], *p* < 0.001, adjusted *R*^2^ = 0.245, $\eta_{p}^{2}$ = 0.085; *B* = 0.166, 95% CI [0.060, 0.272], *p* < 0.01, adjusted *R*^2^ = 0.423, $\eta_{p}^{2}$ = 0.051; *B* = 0.273, 95% CI [0.148, 0.398], *p* < 0.001, adjusted *R*^2^ = 0.525, $\eta_{p}^{2}$ = 0.096; and *B* = 0.217, 95% CI [0.113, 0.320], *p* < 0.001, adjusted *R*^2^ = 0.101, $\eta_{p}^{2}$ = 0.088, respectively). The F-scale (extent that respondents associate negative characteristics with being fat) significantly predicted the perceived suitability of the Normal Weight Male, Obese Weight Male, and Obese Weight Female (*B* = -1.60, 95% CI [-3.1, -0.132], *p* < 0.05, adjusted *R*^2^ = 0.101, $\eta_{p}^{2}$ = 0.026; *B* = -3.53, 95% CI [-5.26, -1.80], *p* < 0.001, adjusted *R*^2^ = 0.51, $\eta_{p}^{2}$ = 0.033; *B* = -2.50, 95% CI [-4.53, -0.48], *p* < 0.05, adjusted *R*^2^ = 0.51, $\eta_{p}^{2}$ = 0.033, respectively).

## Discussion

The current study examined whether obese people are discriminated against when applying for employment. Overall, the current study findings provide further evidence of obesity discrimination in the hiring process for employment. First the findings demonstrated that obese candidates were discriminated against when applying for work compared to normal weight candidates and when the weight status of the candidate was not revealed. These findings are in line with previous reports of obesity discrimination in the hiring process of employment (e.g., Bartels and Nordstrom, 2013; Flint and Snook, 2014; Flint et al., 2015a). This study goes beyond previous work investigating the impact of weight status on recruitment (e.g., Bartels and Nordstrom, 2013) by examining differences in perceived suitability between candidates whose weight status is revealed compared to when it is not revealed. In doing so the current study has demonstrated that when weight status is not revealed, candidates are perceived as more suitable for employment than obese candidates. This effect was observed for both males and females.

Second, the findings demonstrated that obese candidates were evaluated as less suitable across all four workplaces of different physical demand, in particular by participants from heavy manual workforces. This finding demonstrates that irrespective of the physical demand of a job, obese candidates are perceived as less suitable for employment compared with normal weight candidates and when the weight status of the candidate is not revealed. It is likely that stereotypes of obese people as less physically capable and slothfulness (Puhl et al., 2008; Sawbridge and Fitzgerald, 2009) have contributed to this finding.

Third, the current study findings demonstrate that when examining whether the gender of the candidate impacts perceived suitability for work, female candidates were perceived as less suitable across all photo conditions compared to male candidates. Previous research has reported gender differences in perceptions of obesity (e.g., Flint et al., 2016) and that obese female candidates are assessed less favorably than obese males. For example, in a study examining the impact of a defendant’s weight status on perceptions of guilt, Schvey et al. (2013) reported that obese females were more likely to be adjudged as guilty compared to obese males. In addition to demonstrating that overall females compare less favorably to males when applying for work, the current study demonstrates that obese females are perceived as less suitable than obese males across workforces of differing physical demand. For example, obese female candidates were perceived as less suitable for the heavy manual job compared to obese male candidates.

More generally, the current study adds to increasing evidence of obesity discrimination. Given the increasing prevalence of obesity, and thus, greater numbers of overweight and obese candidates, the current study findings require consideration at policy level to ensure all candidates, irrespective of weight status, have equal opportunities for employment. The findings suggest that guidelines for workplace recruitment where weight status is not revealed is warranted. Obesity discrimination needs to be recognized as a rising issue and appropriate legislation has to be regulated, and thus, modification to current UK and EU legislation is required. The current study also demonstrates that irrespective of weight status, females are assessed as less suitable for work across all four workforce groupings based on the physical demands of the job. As such, it might also be suggested that policy development might also consider the removal of gender identification from workplace recruitment. Thus, workplace applications and CVs where gender and appearance are not identified appear to be an appropriate step that leads to a less discriminative process of employment. These results are of particular importance for countries where a photo is required on a CV, such as Spain (Recruitment Spain, 2015) and China (Job Era, 2015).

Finally, in comparison previous research (Bartels and Nordstrom, 2013) examining obesity discrimination in recruitment for work, it might be argued that the current study provides a more realistic design to that of real workplace recruitment. The current study required participants to assess a range of candidates for employment rather than assessing only one candidate’s suitability, and thus more synonymous with real recruitment selection. Furthermore, all participants in the current study had previous experience of recruitment, compared to only 45% of participants in Bartels ad Nordstrom’s study.

The current study is not without limitations. One limitation was the sampling strategy which did not account for gender and BMI. This resulted in uneven amounts of males and females across the four workforce groups, particularly in the heavy manual workplace. There was also a left skew of BMI where overall the sample was slightly overweight. Previous research (e.g., Flint et al., 2015b) has reported differences in anti-fat attitudes based on gender and BMI. Another potential limitation of the study is that whilst all participants were fluent English speakers, the stimuli words used in the IAT might not have been familiar words for all participants impacting response latency. However, IAT scores in the current study are similar to those reported in previous research (e.g., Flint et al., 2015b). Despite this, future cross country research examining implicit attitudes could examine familiarity with stimuli to ensure this potential limitation is avoided. Finally, the construct validity of the IATs has been question. For example, Oswald et al. (2013) conducted a meta-analysis to examine the predictive validity of the IAT and explicit measures as measures of discrimination. Oswald et al. (2013) questioned the performance of the IATs suggesting that they were no stronger than explicit measures. Whist further research is required that sheds light on the validity of IATs, our findings show that anti-fat attitudes are evident on an implicit and explicit level, and that obese candidates are significantly discriminated in recruitment for employment compared to normal weight candidates and when the weight status of the candidate is not revealed. Despite the potential limitations identified, this research has raised some important questions and areas for future research. The workplace environment has a number of impacts such as work satisfaction and productivity. With increasing reports of obesity discrimination in the workplace, future research examining why and in what ways obese people are discriminated whilst in the workplace is warranted. This research should aim to identify both verbal and non-verbal behavior that to the authors’ knowledge is yet to be understood.

## Conclusion

The current study provides evidence of obesity discrimination in the hiring process for employment, where across four workplaces that vary based on the physical demands of the job, obese candidates were perceived as less suitable compared with normal weight candidates and when the weight status of the candidate was not revealed. The study goes beyond previous research examining perceived suitability of obese candidates, using a more valid design whilst addressing methodological shortcomings of previous research. The study demonstrates that gender and weight status impact judgments of suitability for work and that the more physically demanding the job, the more likely it is that obese candidates compared to normal weight or candidates where weight status is not revealed are to less favorable assessments of suitability, and that females compared to males are judged as less suitable for work. Obese female candidates were judged as the least suitable for work, and thus, hold implications for the success rate in the hiring process and therefore unemployment of obese females. The findings contribute to growing calls for policy development to address this growing concern.

## Author Contributions

All authors were involved in the conceptual design of the study. SF and SC contributed to the literature review. SF developed the online IAT and the explicit questionnaires. SF, MČ, SC, and VI contributed to the participant recruitment. SF, MČ, and CZ contributed to the analysis and interpretation of data. All authors read and approved the final manuscript.

## Conflict of Interest Statement

The authors declare that the research was conducted in the absence of any commercial or financial relationships that could be construed as a potential conflict of interest.
